# A Patient-Centered Perspective on Changes in Personal Characteristics After Deep Brain Stimulation

**DOI:** 10.1001/jamanetworkopen.2024.34255

**Published:** 2024-09-18

**Authors:** Amanda R. Merner, Thomas W. Frazier, Paul J. Ford, Brittany Lapin, Joshua Wilt, Eric Racine, Natalie Gase, Essence Leslie, Andre Machado, Jerrold L. Vitek, Cynthia S. Kubu

**Affiliations:** 1 Center for Neurological Restoration, Cleveland Clinic, Cleveland, Ohio; 2 Center for Bioethics, Harvard Medical School, Boston, Massachusetts; 3 Department of Psychology, John Carroll University, University Heights, Ohio; 4 Department of Pediatrics, SUNY Upstate New York, Syracuse; 5 Department of Psychology, SUNY Upstate New York, Syracuse; 6 Center for Bioethics, Cleveland Clinic, Cleveland, Ohio; 7 Department of Neurology, Cleveland Clinic Lerner College of Medicine of Case Western Reserve University, Cleveland, Ohio; 8 Department of Quantitative Health Sciences, Lerner Research Institute, Cleveland Clinic, Cleveland, Ohio; 9 Center for Outcomes Research and Evaluation, Neurological Institute, Cleveland Clinic, Cleveland, Ohio; 10 Department of Psychological Sciences, Case Western Reserve University, Cleveland, Ohio; 11 Montreal Clinical Research Institute, Montreal, Quebec, Canada; 12 Department of Medicine, Université de Montréal, Montreal, Quebec, Canada; 13 Department of Medicine, McGill University, Montreal, Quebec, Canada; 14 Department of Neurology, University of Minnesota, Minneapolis

## Abstract

**Importance:**

Deep brain stimulation (DBS) results in improvements in motor function and quality of life in patients with Parkinson disease (PD), which might impact a patient’s perception of valued personal characteristics. Prior studies investigating whether DBS causes unwanted changes to oneself or one’s personality have methodological limitations that should be addressed.

**Objective:**

To determine whether DBS is associated with changes in characteristics that patients with PD identify as personally meaningful.

**Design, Setting, and Participants:**

This cohort study assessed changes in visual analog scale (VAS) ratings reflecting the extent to which patients with PD manifested individually identified personal characteristics before and 6 and 12 months after DBS at a large academic medical center from February 21, 2018, to December 9, 2021. The VAS findings were tailored to reflect the top 3 individually identified personal characteristics the patient most feared losing. The VASs were scored from 0 to 10, with 0 representing the least and 10 the most extreme manifestation of the trait. Change scores were examined at the individual level. Content analysis was used to code the qualitative data. Qualitative and quantitative analyses were performed from January 12, 2019 (initial qualitative coding), to December 15, 2023.

**Exposure:**

Deep brain stimulation.

**Main Outcomes and Measures:**

The primary outcome variable was the mean VAS score for the top 3 personal characteristics. The secondary outcome was the incidence of meaningful changes on the patients’ top 3 characteristics at the individual level.

**Results:**

Fifty-two of 54 dyads of patients with PD and their care partners (96.3%) were recruited from a consecutive series approved for DBS (36 patients [69.2%] were male and 45 care partners [86.5%] were female; mean [SD] age of patients, 61.98 [8.55] years). Two patients and 1 care partner were lost to follow-up. Increases in the mean VAS score (indicative of greater manifestation of [ie, positive changes in] specific characteristics) were apparent following DBS for ratings of both the patients (Wald χ^2^ = 16.104; *P* < .001) and care partners (Wald χ^2^ = 6.746; *P* < .001) over time. The slopes of the changes for both the patient and care partners were correlated, indicating agreement in observed changes over time. The individual level analyses indicated that scores for most patients and care partners remained the same or increased.

**Conclusions and Relevance:**

In this cohort study, participants reported greater (more positive) manifestations of individually identified, valued characteristics after DBS. These findings may be relevant to informing decision-making for patients with advanced PD who are considering DBS.

## Key Points

**Question:**

Is deep brain stimulation (DBS) associated with unwanted changes in personally valued characteristics among patients with Parkinson disease (PD)?

**Findings:**

This cohort study of 49 patients with PD and their care partners studied changes in visual analog scale ratings reflecting the personal characteristics that patients most feared losing. Scores of the patients and care partners on the visual analog scales increased over time, reflecting greater manifestations of (ie, positive changes in) valued characteristics following DBS.

**Meaning:**

These findings may be relevant to informing decision-making for patients with advanced PD who are considering DBS.

## Teaser

This cohort study addresses concerns about loss of valued personal characteristics following deep brain stimulation for Parkinson disease among dyads of patients and their care partners.

## Introduction

Parkinson disease (PD) is a disorder with motor, cognitive, autonomic, and neuropsychiatric symptoms reflecting changes involving multiple neurotransmitters and brain regions.^1,2,3^ Similarly, motor, cognitive, autonomic, and neuropsychiatric symptoms may also arise secondary to treatment.^2^ Neurobehavioral changes such as depression or dysthymia, anxiety, and apathy have been documented following deep brain stimulation (DBS), with reports showing improvements and worsening of neuropsychiatric symptoms.^4,5^ In addition, early reports commented on changes in interpersonal relationships and self-image, including changes based on qualitative observations.^6,7^ Gisquet^8^ and others have stated that DBS can result in unwanted changes in identity,^8,9^ self,^10,11^ or personality^12^ based on case reports or retrospective interviews with patients who underwent DBS. These findings provide valuable insight from individual patients’ experiences; however, most studies were retrospective and relied on convenience samples, which limits the generalizability of the qualitative data and could result in biased interpretation. Further, the early publications^12,13^ generally did not consider neurobehavioral symptoms associated with the disease, ongoing neurodegenerative changes, or adverse effects of treatment. A handful of studies^14,15,16,17^ examined behavioral changes following DBS using standard tests, most of which focused primarily on measures examining pathological changes (eg, apathy, disinhibition). Those quantitative findings are similarly mixed, with some reporting greater pathology per the measures used^14^ and other studies reporting improvements.^15,16,17^ Most of those quantitative studies consisted of small convenience samples and relied on retrospective ratings of pre-DBS personality, often extending several months to years following surgery, both of which may limit the generalizability and validity of the data.

Despite the methodological limitations outlined above, the concern that DBS can result in unwanted changes has entered the public dialogue^18^ and may affect patient decisions to pursue DBS and potentially contribute to stigma.^19,20^ It is ethically important to address these ongoing concerns to provide appropriate care to patients and better inform public communication. Our study sought to capture the first-person perspectives and qualitative richness evident in the smaller case series, while using a quantitative, prospective approach in a large sample. To accomplish this goal, we prospectively studied a cohort of patients who were candidates for DBS (n = 52). The patients identified the top 3 personal characteristics they most feared losing prior to surgery (given the focus on unwanted, negative changes in the literature). Patients and care partners indicated the extent to which the patient manifested those personally identified characteristics using visual analog scales (VAS). We prospectively assessed changes on the VAS scores reflecting the characteristics identified prior to surgery and at 2 times after DBS. Our method was novel in that we focused on those personal characteristics that patients most feared losing; thus, our outcomes reflect patients’ values.

## Methods

### Study Design and Participants

In this cohort study, we recruited a consecutive series of 58 patients approved for DBS surgery to treat motor symptoms of PD as standard of care at the Cleveland Clinic from February 21, 2018, to December 9, 2019, with follow-up completed February 10, 2021. Although delayed by the COVID-19 pandemic, qualitative and quantitative analyses were performed from January 12, 2019 (initial qualitative coding), to December 15, 2023. All patients undergoing DBS completed a comprehensive, multidisciplinary evaluation including neurological, neurosurgical, neuropsychological, and psychological assessments. Inclusion criteria included age of 21 years or older, diagnosis of idiopathic PD by a board-certified movement disorder neurologist, native English speaker, availability of a care partner, no evidence of severe visual or hearing impairments, no diagnosis of dementia, no comorbid neurological disorder or history of neurological injury, and no prior DBS procedure or neurosurgical treatment for PD. Four of the 58 patients approached did not have a care partner and, thus, did not meet our inclusion criteria. One participant declined participation due to illness and another failed to attend the research appointment. Our final sample included 52 patients and their care partners, resulting in an overall recruitment rate of 96.3% (52 of 54 patients who met all inclusion criteria). Our study sought to recruit patients who reflected the demographic characteristics of our broader clinical population, including race. All participants provided written informed consent, and ethical approval for all study-related procedures was obtained from the Cleveland Clinic Institutional Review Board. This study meets all Strengthening the Reporting of Observational Studies in Epidemiology (STROBE) guidelines.

### Procedures

Patients and care partners completed separate research sessions, including a semistructured interview, rating scales (eMethods 1 in Supplement 1), and standard personality measures (ie, Iowa Scales of Personality Change, Frontal Systems Behavior Scale, and the NEO).^21,22,23^ At the baseline (presurgery) session, patients were asked to identify the top 3 personal characteristics they most feared losing. Patients and care partners rated the extent to which the patient demonstrated those top 3 characteristics using the patient’s initial description on the VAS, with higher scores representing the greatest manifestation of that characteristic. Patients and care partners also rated the patient’s global self on a VAS, with higher scores representing the extent to which the patient was unchanged with respect to their thoughts, feelings, and actions since the first symptom of PD. The VASs are widely used in clinical settings and are reasonable methods to assess subjective experiences.^24^ Participants completed these 4 VASs prior to surgery and at postoperative months 6 and 12, allowing for a prospective quantitative assessment of changes in the top 3 personal characteristics identified at baseline and a global measure of self.

### Outcomes

Our primary outcome variable for this study was the mean of the VAS ratings for the top 3 characteristics identified by the patients undergoing DBS. Our secondary outcome was the incidence of meaningful changes on the patients’ top 3 characteristics at the individual level, which was operationally defined as change scores (characteristic postoperative month 6 VAS score minus the baseline VAS score) of at least 1 SD. Additional outcomes included the care partners’ mean VAS, individual change scores based on the care partners’ ratings, and the global self-assessed VAS scores of the patients and care partners. We also collected the Unified Parkinson’s Disease Rating Scale III (UPDRS-III) motor subscale data for the patients.

### Qualitative Analyses

We adopted a content analysis approach to examine the raw qualitative data and create qualitative codes that described the individual characteristics identified by participants in the interview data. The qualitative codes were derived from a larger study. The 32 individual codes were recoded into broader, overarching categories based on an exploratory factor analysis to simplify data presentation (eMethods 2 in Supplement 1).

### Quantitative Analyses

Descriptive statistics, correlations, parallel process growth models, and generalized estimating equations (GEE) were used to describe the data and relationships between variables and groups (ie, patients and care partners) and to examine changes over time. Given the concerns in the literature that DBS results in unwanted and negative behavioral changes, we did not correct for repeated analyses to maximize the possibility of identifying any potential negative changes. We also examined changes at the individual patient level using change scores with meaningful change, operationally defined as change scores of 1 SD or greater. Pearson correlation coefficients were used to examine the association between UPDRS-III motor improvement and change in personality over time.

### Statistical Analysis

A sample size of 52 patient–care partner dyads provided good statistical power (≥0.80) to detect medium-to-large correlations (*r* ≥ 0.34) between patient and care partner scores and external correlates (1-tailed α = .05). To estimate power to detect changes over time in personally identified characteristic ratings, a repeated-measures analysis of variance was used analogous to the GEEs to evaluate change. This model assumed only modest correlations between repeated measures (*r* = 0.20) as a more conservative estimate. Using this model, statistical power was expected to be very good or better (≥0.87) to detect a medium main effect size (*F* = 0.25; Cohen *d* = 0.50) representing changes over time in the ratings.

Parallel process growth modeling was used to examine correlations between changes in ratings by patients and care partners (correlation of slopes). To examine the power to detect significant slope correlations in this model, a simulation study (*K* = 1000 samples [N = 52]; 1-tailed α = .05) was conducted using the same data characteristics as the planned analysis (2 parallel process measures at 3 equidistant times). Results indicate good power (≥0.83) to detect a medium-to-large correlation between patient and care partner slopes (standardized path coefficient = 0.35). One-tailed testing was used for all analyses based on specific directional estimates and the desire to be maximally sensitive to any potential changes in personality scores, with *P* < .05 indicating statistical significance.

## Results

### Participant Characteristics

Table 1 provides demographic and disease-related data for all participants initially recruited. Of the 52 dyads, 16 patients (30.8%) were female and 36 (69.2%) were male; whereas 45 care partners (86.5%) were female and 7 (13.5%) were male. Mean (SD) age of patients was 61.98 (8.55) years and of care partners, 57.35 (13.12) years. In terms of self-reported race, 2 patients (3.8%) and 1 care partner (1.9%) were Asian; 2 patients (3.8%) and 1 care partner (1.9%), Black; and 48 patients (92.3%) and 50 care partners (96.2%), White.

**Table 1.** Patient and Care Partner Demographic and Disease Data^a^

Variable	Patients (n = 52)	Care partners (n = 52)
Sex		
Female	16 (30.8)	45 (86.5)
Male	36 (69.2)	7 (13.5)
Age (SD), y	61.98 (8.55)	57.35 (13.12)
Race^b^		
African American or Black	2 (3.8)	1 (1.9)
Asian	2 (3.8)	1 (1.9)
White	48 (92.3)	50 (96.2)
Educational level, mean (SD), y	15.13 (2.63)	15.13 (2.42)
Time since PD diagnosis, mean (SD), y	9.18 (5.43)	NA
Time known patient, mean (SD), y	NA	34.38 (14.98)
Relationship to patient		
Spouse	NA	34 (65.4)
Son or daughter	NA	8 (15.4)
Friend	NA	2 (3.8)
Other	NA	8 (15.4)
UPDRS-III score, mean (SD)^c^		
Preoperative, with medication	18.15 (10.57)	NA
Preoperative, without medication	41.74 (12.89)	NA
Postoperative, with medication and stimulation	11.89 (8.54)	NA
Surgical target		
Subthalamic nucleus	45 (90.0)	NA
Globus pallidus interna	5 (10.0)	NA
Surgical side^d^		
Bilateral	43 (84.3)	NA
Left	5 (9.8)	NA
Right	3 (5.9)	NA

Abbreviations: NA, not applicable; UPDRS-III, Unified Parkinson’s Disease Rating Scale III.

^a^ The descriptive data include all participants initially recruited since our analyses incorporated an intention-to-treat approach. Unless otherwise indicated, data are expressed as No. (%) of patients and care partners.

^b^ Participants self-reported race.

^c^ Includes 42 patients. Scores range from 0 to 132, with higher scores indicating greater motor disability.

^d^ One patient did not have surgery.

Following preoperative semistructured interviews, 2 patients were lost to follow-up. One patient did not move forward with surgery. A second had their device implanted but decided to complete programming at another institution. Finally, 1 care partner was unavailable for follow-up although the patient participated in the postoperative research appointments. This yielded a final sample of 49 patient–care partner dyads who completed all 3 assessments.

### Qualitative Findings

The content analysis yielded a set of 32 descriptive codes based on the care partners’ descriptions of the patients’ personal characteristics (eMethods 2 in Supplement 1). Excellent interrater reliability was observed (κ = 0.80). An exploratory factor analyses yielded an 8-factor structure comprised of the factors listed in Table 2.^25^ The individual codes were recoded using these 8 overarching factors to simplify data presentation (eTable 1 in Supplement 1). Where appropriate, we attempted to label the factors with terms commonly used in the psychological literature.

**Table 2.** Factors Derived From Original Content Analysis

Factor	Original codes
Prosocial	Loving and/or caring, compassion and/or kindness, generous
Physical^a^^,^^b^	Basic motor functions, athletic, or above-average physical abilities
Cognitive	Basic cognitive abilities, above-average cognitive skills, intellectual curiosity
Self-regulation	Conscientious, independent, leader and/or decisive, hard worker, meaningful work, fortitude
Mixed character virtues^c^	Integrity, loving fidelity and/or commitment to family or friends, tolerance, humility, quiet and/or shy, faith in God
Positive emotionality	Social and/or friendly, humor, optimism, happy, high energy and/or vitality, confidence
Negative internalizing emotionality	Anxious, depressed
Negative externalizing emotionality	Irritable and/or mean, inflexible, competitive

^a^ Inclusion of physical personality characteristics is inconsistent with some accounts of personality but not all^25^; regardless, physical characteristics were reported as being important components of participants’ views of personality.

^b^ Originally, there was also a code of other for physical that included sexuality and appearance but that did not load on any of the factors.

^c^ We recognize that some of the personality codes that loaded on this factor may not correspond to classically defined virtues and that other factors (eg, prosocial, self-regulation) may contain personality codes traditionally included in classic discussions of virtue. We opted to use the descriptive term *mixed character virtues*, as many of the codes in this factor correspond to what the public would characterize as virtuous behavior.

**Figure 1.**
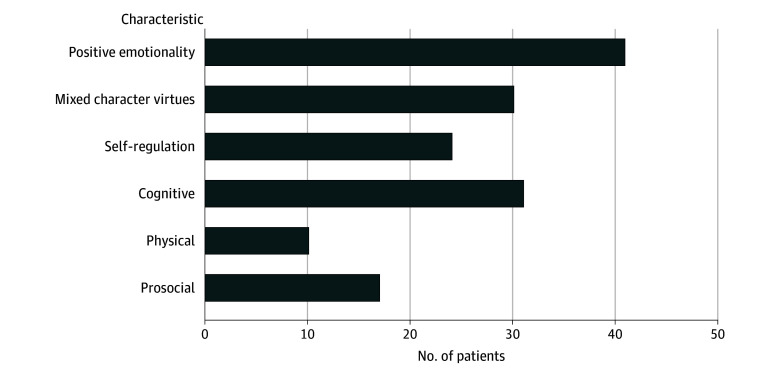
Number of Patients’ Top 3 Personality Characteristics by Personality Factor Mixed character virtues indicate the codes that correspond to what the public would characterize as virtuous behavior.

All patients identified the top 3 characteristics they feared losing the most. Across all 3 top ranked characteristics, characteristics related to positive emotionality accounted for most of the patients’ responses (Figure 1).

### Primary Outcome

**Figure 2.**
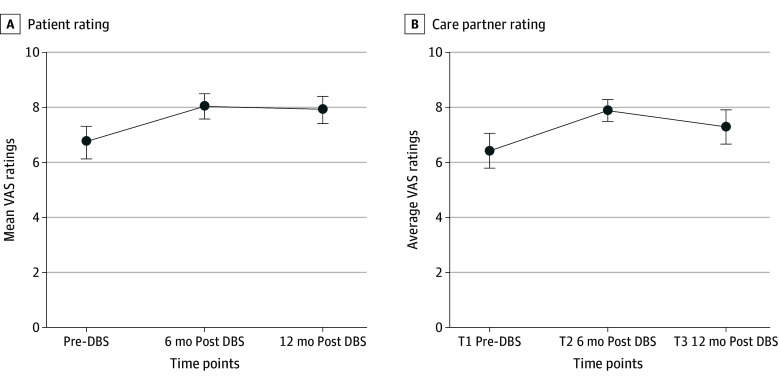
Top 3 Personality Characteristics Mean Visual Analog Scale (VAS) Scores Over Time Higher scores indicate greater manifestation of the characteristic. Error bars represent 95% CIs. DBS indicates deep brain stimulation.

The patients’ VAS scores were positively correlated (*r* range, 0.357-0.649; *P* ≤ .01 for all). Streiner and Norman^26^ have provided guidance that short scales with few items should use correct item total correlations of 0.30 or greater as evidence of good scale reliability. Intraclass correlations were also run to examine test-retest reliability of the VAS scores from postoperative months 6 to 12. The test-retest reliability for the mean score for characteristics 1 to 3 for patients was excellent (*r* = 0.828).^27^ We used GEEs to examine group changes over time on the patients’ mean VAS scores for the top 3 characteristics (Figure 2). Those data indicate that the mean rating of all 3 characteristics increased over time from the perspectives of the patients (Wald χ^2^ = 16.104; *P* < .001) and care partners (Wald χ^2^ = 6.746; *P* < .001) (eTable 2 in Supplement 1 provides the results for the patients’ global VAS and the care partners’ mean VAS and global VAS scores; eFigures 1 and 2 in Supplement 1 provide the data for the individual characteristics scores across time).

Parallel process growth models were also computed to assess whether the patient and care partner scores showed consistent change. In these models, patient and care partner slopes were positively correlated for the mean top 3 characteristics (*r* range, 0.30-0.66) and global score (*r* range, 0.44-0.48) illustrating the concordance between patients’ and care partners’ observations of changes over time (eFigure 3 in Supplement 1).

Finally, we evaluated the association with the UPDRS-III. There was a significant improvement in UPDRS-III motor scores following DBS. We also examined the association between change in the VAS ratings and change in the UPDRS-III overall motor scores (pre-DBS score without medication minus 6-month score with medication and stimulation). None of the correlations were significant (*r* range, −0.121 to 0.261; *P* > .05 for all) suggesting that the changes in the VAS scores were unrelated to changes in motor symptoms.

### Secondary Outcome: Individual-Level Analyses

The data were analyzed for both the patient and care partner participants’ ratings on an individual basis to determine the incidence of meaningful changes on each of the top 3 characteristics. Meaningful change occurred for 27 of 49 patients (55.1%) (43 changes). Of those, 5 change scores were negative, suggesting reduced manifestation of that characteristic, in 4 patient participants. The remaining 38 meaningful change scores were positive (88.4% in 23 patients) indicating that the patient demonstrated that characteristic more following surgery. The care partner data were similar with 42 changes, of which 5 were negative changes (11.9%) in 4 patients and 37 were positive changes (88.1%) in 21 patients (Table 3).

**Table 3.** Number of Participants Meeting Criteria for Meaningful Change at the Individual Level

Factor	No. of participants	
	Patient	Care partner
**Reduced scores (reflecting less of the characteristic)**		
Prosocial	0	1
Cognitive	2	2
Physical	1	0
Self-regulation	1	1
Mixed character virtues	1	0
Positive emotionality	0	1
Negative internalizing emotionality	0	0
Negative externalizing emotionality	0	0
**Higher scores (reflecting more of the characteristic)**		
Prosocial	5	5
Cognitive	5	7
Physical	7	4
Self-regulation	7	7
Mixed character virtues	7	5
Positive emotionality	7	7
Negative internalizing emotionality	0	0
Negative externalizing emotionality	0	0

## Discussion

This study used a cohort design to prospectively examine changes in personally identified characteristics following DBS to treat motor symptoms of PD in 49 patient–care partner dyads. We adopted an atheoretical approach and used the patients’ individually defined and prioritized personal characteristics to examine changes over time. Many of the responses did not reflect prototypical traits or behaviors reflected in standard measures, but instead reflect patient-specific values related to a variety of individual characteristics. Our results suggest that for most patients, DBS does not result in negative changes in the individually identified personal characteristics that patients most feared losing. The data indicate that for some patients, DBS resulted in increased manifestation of the valued characteristics they would be most unwilling to lose such that mean scores approached their retrospectively identified historical scores (eFigure 4 in Supplement 1). This was true when we assessed both the patients’ and care partners’ responses, which showed concordance in their responses over time. It is unlikely that recency of the surgery or cognitive dissonance played a significant role in participants’ higher scores after surgery, given the postoperative assessments occurred at 6 and 12 months following surgery. Notably, the changes in characteristics were unrelated to changes in the UPDRS-III motor score following DBS. This is not surprising, since most of the highly ranked characteristics were not physical characteristics. We also examined individual changes in ratings over time, and those findings illustrate that meaningful reductions of valued characteristics were apparent in 4 patients, whereas 23 of the 49 patients reported higher scores in 1 or more characteristics such that they manifested the valued characteristics more following DBS. Similar findings were apparent in the care partner VAS scores.

Neurobehavioral changes can occur following DBS for the treatment of motor symptoms of PD due to stimulation, lead location, medication changes, disease progression, and psychosocial issues^2,3,4,5,6,7,12,28^ The study by Agid and colleagues^6^ was one of the first to highlight the disconnect between significant improvements in motor symptoms following DBS and dissatisfaction in other aspects of the patient’s life. The authors explicitly stated that “the difficulty in social integration experienced by our operated patients results, not directly from a modification of the patients’ personality, but rather indirectly from a difficulty of reintegrating into the sociofamilial and professional environment.”^6^ The theme of the burden of normality has been discussed in the functional neurosurgery literature before.^29,30,31^ As noted, prior studies raised concerns about negative and unwanted changes,^8,9,10,11,12^ yet careful review indicates that in many of these reports, the changes reflect a mix of positive and negative changes.^10,11,32^ Thomson and colleagues^32^ note that DBS was viewed as restorative to personality and self by some in their final sample of 9 patient–care partner dyads studied prospectively with qualitative interviews. If unwanted changes were apparent, they tended to be transient and were often attributed to adverse effects of stimulation, disease progression, and changes associated with symptom improvement and/or were viewed as relatively minor given the benefits.^32^ In short, prior studies suggest there can be some changes in personality, self, identity,^8,9,10,11,12,32^ and/or sociofamilial relationships^6,7^ following DBS for the treatment of motor symptoms of PD. However, the prevalence is uncertain, and the causes are multifactorial and may include factors unrelated to DBS. To our knowledge, our study is the first to quantitatively examine changes in personally valued characteristics prospectively based on patient’s understandings and values in a consecutive series of patients. Second, our data show that behaviors related to positive emotions were the characteristics patients identified as those they most feared losing. The importance of positive emotionality was followed by characteristics related to cognition and mixed character “virtues” (term indicates many of the codes in this factor correspond to what the public would characterize as virtuous behavior) with smaller proportions in self-regulation, prosocial, and physical characteristics.

These findings have implications beyond the use of DBS to treat motor symptoms of PD. Narratives that highlight negative, unwanted changes using language such as personality, self, or identity^8,9,10,11,18^ can lead to further stigmatization of patients with brain implants and dissuade patients from seeking neuromodulation treatments that are widely considered effective.^20^ Our data may reduce some of the stigma associated with brain implants. These findings also support the development and inclusion of outcome measures that reflect patients’ values and understandings.^33,34^ The inclusion of outcome measures based on patients’ understandings may have greater ecological validity and lead to improved communication, informed consent processes, and better clinical trial design.

### Limitations

There are several limitations to these findings. We intentionally prioritized the individually identified and valued personal characteristics of patients and care partners; thus, our findings must be interpreted in that context and may not reflect standard theories or measures of related constructs or all aspects of the patient’s personality or sense of self. This latter limitation is somewhat mitigated by the inclusion of a global VAS that incorporated all aspects of the patients’ pre-PD sense of self and would presumably capture a broad range of characteristics from the perspectives of both the patients and care partners. Second, our data were collected at 1 surgical center on a sample with limited diversity. This work needs to be extended to other patient and demographic groups, as it is possible that sociocultural and/or disease variables will impact responses. Our sample size was also relatively small for a quantitative study (albeit large for a qualitative investigation). We corrected for that by considering effect sizes and conducting individual participant analyses using change scores to examine changes over time. Our individual change analyses relied on a simple statistical criterion (ie, SD) that does not necessarily correspond to clinical meaningfulness. As noted, we chose not to correct for multiple comparisons to be especially sensitive to any perceived changes. Finally, our data need to be replicated, and the preliminary factor structure identified in our data may change with larger and more diverse samples.

## Conclusions

In this cohort study, we found that DBS did not result in unwanted changes in valued personal characteristics at the group level; in contrast, increased manifestations of valued personal characteristics were apparent. Our individual participant analyses revealed that negative changes were apparent in 4 patients. Most patients and their care partners indicated no change or increased manifestation of valued personal characteristics following DBS equally spread across the prosocial, cognitive, physical, self-regulation, mixed character virtues, and positive emotionality factors. Our findings illustrate the importance of prospective assessments in examining changes in personal characteristics following DBS in a consecutive cohort of patients.

## Notes

Supplement 1. **eMethods 1.** Interview Protocol **eMethods 2.** Qualitative Methods and Results **eTable 1.** Factors and Related Personal Characteristic Codes **eTable 2.** Changes in Mean Top 3 and Global VAS Ratings Over Time **eFigure 1.** Mean Patient VAS Scores for Top 3 Characteristics Over Time With 95% CI **eFigure 2.** Mean Care Partner VAS Scores for Top 3 Characteristics Over Time With 95% CI **eFigure 3.** Association Between Patient and Care Partner Participants’ Scores Over Time Across All 3 Characteristics **eFigure 4.** Mean Top 3 Characteristic Scores With 95% CI

Supplement 2.
Data Sharing Statement
