# Supplementary material for: A Patient-Centered Perspective on Changes in Personal Characteristics After Deep Brain Stimulation
Source: JAMA Netw Open. 2024 Sep 18;7(9):e2434255. doi: 10.1001/jamanetworkopen.2024.34255 (PMC11411387; doi:10.1001/jamanetworkopen.2024.34255)
Supplement: Supplement 1. — eMethods 1. Interview Protocol eMethods 2. Qualitative Methods and Results eTable 1. Factors and Related Personal Characteristic Codes eTable 2. Changes in Mean Top 3 and Global VAS Ratings Over Time eFigure 1. Mean Patient VAS Scores for Top 3 Characteristics Over Time With 95% CI eFigure 2. Mean Care Partner VAS Scores for Top 3 Characteristics Over Time With 95% CI eFigure 3. Association Between Patient and Care Partner Participants’ Scores Over Time Across All 3 Characteristics eFigure 4. Mean Top 3 Characteristic Scores With 95% CI [file jamanetwopen-e2434255-s001.pdf]

Supplementary Online Content

Merner AR, Frazier TW, Ford PJ, et al. A patient-centered perspective on changes in personal characteristics after deep brain stimulation. *JAMA Netw Open*. 2024;7(X):e2434255. doi:10.1001/jamanetworkopen.2024.34255

eMethods 1. Interview Protocol
eMethods 2. Qualitative Methods and Results
eTable 1. Factors and Related Personal Characteristic Codes
eTable 2. Changes in Mean Top 3 and Global VAS Ratings Over Time
eFigure 1. Mean Patient VAS Scores for Top 3 Characteristics Over Time With 95% CI
eFigure 2. Mean Care Partner VAS Scores for Top 3 Characteristics Over Time With 95% CI
eFigure 3. Association Between Patient and Care Partner Participants' Scores Over Time Across All 3 Characteristics
eFigure 4. Mean Top 3 Characteristic Scores With 95% CI

This supplementary material has been provided by the authors to give readers additional information about their work.

© 2024 Merner AR et al. *JAMA Network Open*.

eMethods 1. Interview Protocol

We are interested in better understanding any changes you may have observed over the course of your Parkinson's disease in your personality. Psychologists define personality as characteristic patterns of thinking, feeling, and behaving. We are particularly interested in your perceptions regarding changes in who you are as a person.

Please take a few minutes and tell me some of the words that you feel best describe your personality throughout most of your life.

……..

Great. Now, I'd like you to tell me the top five personality traits that you most fear losing.

(*Some participants could not generate five; thus, we required a minimum of three)

Please rank order these characteristics such that #1 is the feature you most fear losing (most important to maintain):

Most 1: ____

2: ____

3: ____

4: ____

Least 5: ____

Trait 1: _________________________

Please place a mark on the line below indicating the extent to which you demonstrate Trait 1 ________________ at the present time. Zero represents the least manifestation of that trait whereas 10 represents the most extreme manifestation of that trait.

© 2024 Merner AR et al. *JAMA Network Open*.

0 ____ 10
Least Most

(Similarly for remaining top characteristics)

……

Global Rating

Please place a mark on the line below indicating how much you feel you are the same person you have always been with respect to your thoughts, feelings, and actions since you noticed your first symptom of Parkinson's disease.

0 ____ 10

Not me at all Completely me

Please tell me why:

eMethods 2. Qualitative Methods and Results

The current study focused on the surgical cohort who was part of a larger study assessing how a large sample of patients with PD and their care partners (n=156 dyads) describe the patients' personality over the course of their lifetime to better capture their understandings of personality. We operationally defined personality in our interviews as characteristic ways of thinking, feeling, and behaving per one of the classic psychological definitions. A subset of transcripts (n = 60, including 20 transcripts from our surgical cohort reflecting the first 10 dyads) was reviewed, and broad themes were identified. Responses to the question related to the patient's personality over their lifetime underwent thematic content analyses to inductively and iteratively identify recurring themes related to participants' personality descriptions. Transcripts were read closely again to identify more nuanced and specific codes that fell within the larger themes.[32] This process resulted in an initial set of 32 codes (e.g., "loving, caring", "happy, optimistic, positive", see eTable 1).

After this coding structure was finalized, two raters independently coded a subset of 60 transcripts (30 patient, 30 care partners from the total sample of 156 surgical participant-care partner dyad transcripts) balanced by interviewer, age, gender, and race/ethnicity. 543 unique descriptors (e.g., "loving", "positive", "happy") were double coded from this data subset. Kappa statistics were computed to determine the inter-rater reliability of the coding structure. Excellent inter-rater reliability was observed (Kappa=.80).

© 2024 Merner AR et al. *JAMA Network Open*.

To simplify data presentation and identify the underlying factor structure of personality as defined by our sample, we utilized an exploratory factor analysis (EFA). The care partner participants' descriptions of the patients' personality (n=156) were coded using the previously identified codes by one coder (CSK). Each excerpt of text (total = 886) from the care partner interviews describing the patient's personality was transformed into a survey item with a four-point Likert scale with "Not at all similar" at one end and "Extremely similar" at the other. Two raters independently rated the similarity of every text excerpt to each of the 32 codes derived from the content analysis using the four-point Likert scale. Interclass correlations were conducted to assess reliability of ratings. Averages for the two raters were then computed for each personality code that was determined to be reliable between raters. Those averages were used as variables in an exploratory factor analysis (EFA) to examine the relationships among the personality codes and identify higher level codes.

The EFA indicated that an eight-factor solution fit the data best. χ^2 (268) = 1776.308, p = .000, RMSEA = 0.08, CFI = 0.865, TLI= 0.750, SRMR = 0.032. Table S1 provides the individual 32 codes derived from the content analysis and the factor with which they loaded highest. As noted in the main text, we attempted to label the factors with terms commonly used in the psychological literature (e.g., internalizing vs externalizing negative emotionality like internalizing vs externalizing disorders). We acknowledge that traditional personality tests do not incorporate physical abilities or characteristics; however, these were characteristics our sample identified in their descriptions of personality and correspond to other considerations of self in which physical (i.e., motor, sensory) characteristics are incorporated.[22] We also recognize that some of the personality codes that loaded on the Mixed Character "Virtues" factor may not correspond to classically defined virtues and that other factors (e.g., Prosocial, Self-Regulation) may contain personality codes traditionally included in classic discussions of virtue. For the purposes of this initial paper, we opted to use the descriptive term Mixed Character "Virtues" as many of the codes in this factor correspond to what the public would characterize as virtuous behavior.

1. **eTable 1.** Factors and Related Personal Characteristic Codes

Broader Factor	Individual Codes
**Prosocial**	1. Loving, caring<br>2. Compassion, kindness, understanding, empathy<br>3. Generous (e.g., "shirt off his back", "very giving", "helpful", generous with time)<br>4. Emotional intelligence (e.g., "awareness", understanding of others' perspectives with specific reference to cognitive

© 2024 Merner AR et al. *JAMA Network Open*.

	understanding others (e.g., intuitive, sensitive to others' feelings
**Physical**	5. Basic physical abilities (e.g., walking, mobility)<br>6. Beyond basic physical abilities/Athletic abilities (e.g., biking Running, "S/he is an athlete"
**Cognitive**	7. Basic cognitive abilities (e.g., memory, processing speed, judgment/poor judgment/impulsivity)<br>8. More than basic cognitive abilities (e.g., "S/he is extremely bright/intellectual/brilliant")<br>9. Intellectual curiosity, Joie de vivre (also includes fascination/interest in hobbies/traveling, anything to do with exploring the world/ideas/activities)
**Self-Regulation**	10. Conscientiousness/reliable/dependable/responsible/organized (e.g., "you can count on him/her", "always there for you")<br>11. Fortitude (e.g., moral strength, strength in the face of adversity, determination to overcome challenges)<br>12. Independent/self-sufficient (e.g., ability to take care of themselves)<br>13. Industrious, hard worker<br>14. Leader, decisive, task-oriented, takes charge/initiative, driven, motivated<br>15. Meaningful work (includes the idea of giving back to society or having a higher purpose in life)
**Mixed Character "Virtues"***	16. Relationship-specific (e.g., "our love", "great stepdad", "best friend", any term that specifically references a relationship and commitment to a relationship<br>17. Humble, humility<br>18. Integrity, honesty, sincere, loyal<br>19. Easy-going, calm, patient, mellow, open-minded regarding other people, non-judgmental<br>20. Quiet, introvert, shy<br>21. Religious/Faith
**Positive Emotionality**	22. High energy, vitality<br>23. Confidence (e.g., self-assured)<br>24. Happy, optimistic, positive<br>25. Humor, funny, jokester (any reference to sense of humor, joking)<br>26. Social/friendly/extravert (e.g., "life of the party", "social Butterfly")
**Negative Internalizing Emotionality**	27. Sad/depressed<br>28. Anxious, worried, nervous
**Negative Externalizing Emotionality**	29. Irritable, grumpy, cranky, mean<br>30. Inflexible, rigid, stuck in their ways, stubborn, close-minded<br>31. Competitive
**Item did not load[a]**	32. Libido/Sexual relationship

[a]This code emerged very rarely and was not cited as a key personality characteristic in the DBS cohort.

© 2024 Merner AR et al. *JAMA Network Open*.

2. **eTable 2.** Changes in Mean Top 3 and Global VAS Ratings Over Time

Variable		Wald χ^2 Time	*p* value	Effect size
**Average of Top Three Characteristics**				
	Patient	16.104	<.001	d=0.6962
	Care Partner	6.746	<.001	d=0.4355
**Global Personality**				
	Patient	25.738	<.001	d=0.9139
	Care Partner	25.414	<.001	d=0.9069

3. **eFigure 1.** Mean Patient VAS Scores for Top 3 Characteristics Over Time With 95% CI

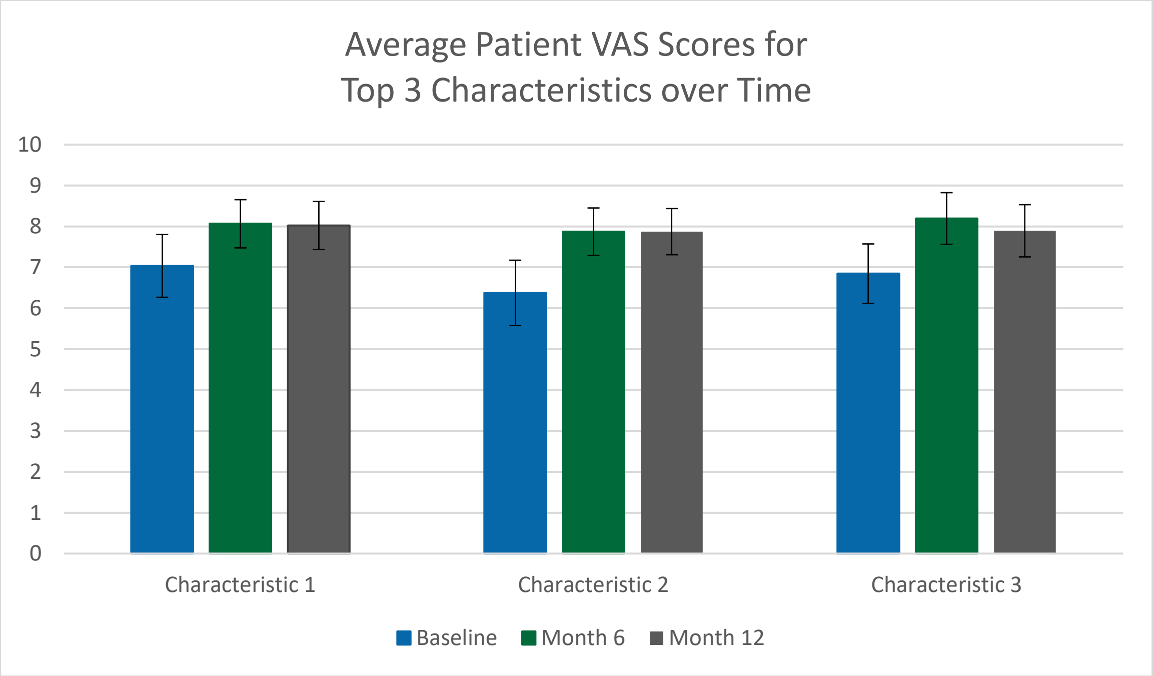

4. **eFigure 2.** Mean Care Partner VAS Scores for Top 3 Characteristics Over Time With 95% CI

© 2024 Merner AR et al. *JAMA Network Open*.

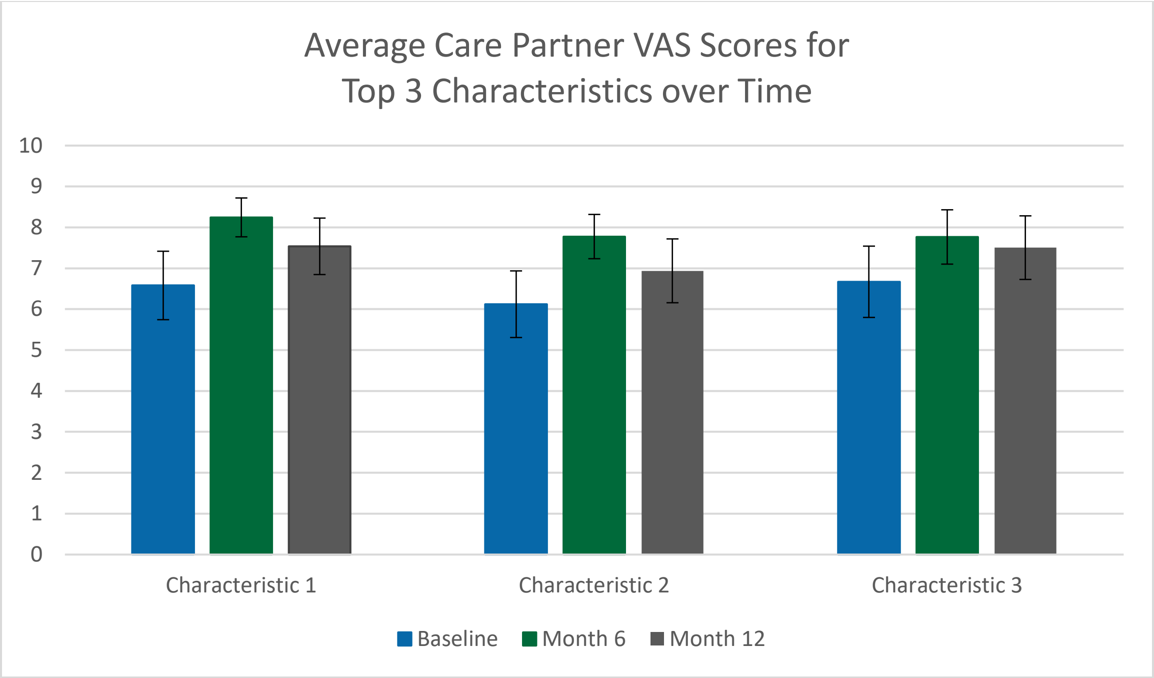

© 2024 Merner AR et al. *JAMA Network Open*.

5. **eFigure 3.** Association Between Patient and Care Partner Participants' Scores Over Time Across All 3 Characteristics

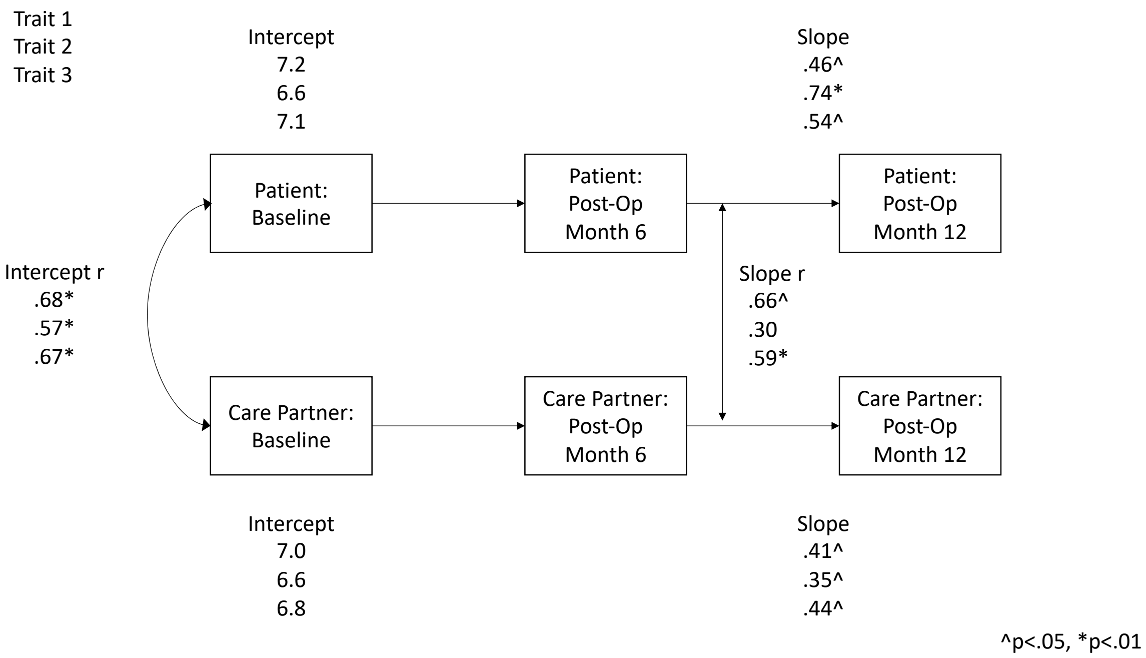

6. **eFigure 4.** Mean Top 3 Characteristic Scores With 95% CI

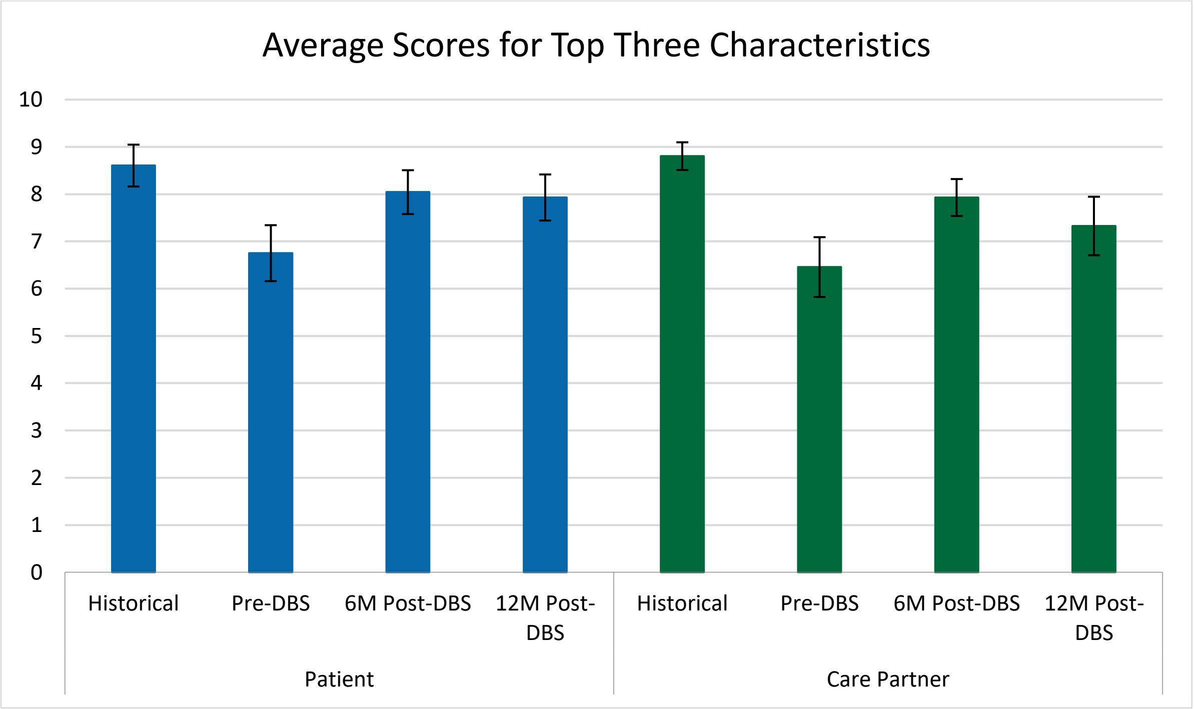

© 2024 Merner AR et al. *JAMA Network Open*.

[a]Historical score is a retrospective rating, all others are prospective

© 2024 Merner AR et al. *JAMA Network Open*.
