# Supplementary material for: A Patient-Centered Perspective on Changes in Personal Characteristics After Deep Brain Stimulation
Source: JAMA Netw Open. 2024 Sep 18;7(9):e2434255. doi: 10.1001/jamanetworkopen.2024.34255 (PMC11411387; doi:10.1001/jamanetworkopen.2024.34255)
Supplement: Supplement 2. — Data Sharing Statement [file jamanetwopen-e2434255-s002.pdf]

Data Sharing Statement

Merner. A Patient-Centered Perspective on Changes in Personal Characteristics After Deep Brain Stimulation. *JAMA Netw Open*. Published September 18, 2024. doi:10.1001/jamanetworkopen.2024.34255

Data
Data available: No

Additional Information
Explanation for why data not available: Do to privacy concerns related to the sensitive nature of some of the qualitative transcripts, we will not provide those raw data but will provide summary qualitative data.
